# P-2120. Clinical significance of 16S rRNA sequencing identification of *Legionella* species

**DOI:** 10.1093/ofid/ofae631.2276

**Published:** 2025-01-29

**Authors:** Laura Steenberge, Joseph McBride

**Affiliations:** UPMC Children’s Hospital of Pittsburgh; Medical Scientist Training Program, University of Wisconsin Medicine and Public Health, Madison, Wisconsin; University of Wisconsin Madison School of Medicine and Public Health, Madison, Wisconsin

## Abstract

**Background:**

Incorporation of 16S rRNA sequencing into clinical diagnostics has improved the ability of clinicians to diagnose fastidious bacteria and facilitates the identification of causative organisms in culture-negative infections. However, its clinical utility is unclear, with estimates of antimicrobial management changes from 16S identification ranging from only 4-15%. *Legionella* species are fastidious bacteria that can be difficult to identify without prior clinical suspicion, and thus can cause culture-negative infections that lead to 16S sequencing. However, *Legionella* are found ubiquitously in aquatic environments and its identification on 16S could represent a clinically significant infection, a nonpathogenic sample isolate, or a contaminant from sample processing or analysis. In this study, we reviewed 16S *Legionella* species results from our institution and determined whether they represented a clinical infection.

**Methods:**

16S results from our institution were queried for *Legionella* species and a retrospective analysis of the resulting 10 cases was conducted. Multiple factors from the cases were assessed including demographics, comorbidities, type of 16S specimen, and antibiotic regimen change in response to 16S results. Additionally, we identified whether the 16S results represented clinically significant infections or contaminants.

**Results:**

70% of the *Legionella* identifications were indicative of a clinically relevant infection. Specimens from a pulmonary source were much more likely to represent a true infection, while identifications from extrapulmonary sources were more likely to be a contaminant. The species of *Legionella* identified on 16S did not impact the likelihood of a true causative agent. The 16S results affected antimicrobial regiments in 60% of the cases and were often the only diagnostic test to point towards *Legionella* as a causative agent.

**Conclusion:**

*Legionella* species identification on 16S can be an important diagnostic clue to the etiology of a patient's infection, but various clinical factors should be taken into account when assessing whether the result represents a true infection or contaminant.

**Disclosures:**

All Authors: No reported disclosures

